# Psychoneurobiological Aspects of Burning Mouth Syndrome and Oral Lichen Planus: A Narrative Review

**DOI:** 10.3390/medicina61081489

**Published:** 2025-08-20

**Authors:** Dora Martić, Ana Glavina, Liborija Lugović-Mihić, Maja Vilibić

**Affiliations:** 1 Department of Oral Medicine, Study of Dental Medicine, School of Medicine, University of Split, 21000 Split, Croatia; doramartic20@gmail.com; 2 Department of Dental Medicine, University Hospital of Split, 21000 Split, Croatia; 3 Department of Dermatovenereology, University Hospital Center Sestre Milosrdnice, 10000 Zagreb, Croatia; liborija@gmail.com; 4 School of Dental Medicine, University of Zagreb, 10000 Zagreb, Croatia; 5 Department of Psychiatry, University Hospital Center Sestre Milosrdnice, 10000 Zagreb, Croatia; maja.vilibic@gmail.com; 6 School of Medicine, Catholic University of Croatia, 10000 Zagreb, Croatia

**Keywords:** anxiety, burning mouth syndrome, central desensitisation, cortisol, depression, neuroimmune interaction, oestrogen, oral lichen planus, psychoneuroendocrineimmune axis, stress

## Abstract

Burning mouth syndrome (BMS) and oral lichen planus (OLP) are two chronic oral diseases/disorders that continue to pose a challenge for conventional diagnosis and treatment. Both diseases do not occur in isolation but rather appear to reflect a broader interplay of psychological, neurological, endocrine, and immunological factors, i.e., complex disorders in interconnected biological and psychological systems. In BMS, patients often suffer from persistent burning sensations without visible lesions, which may be related to altered pain processing, emotional stress, and dysregulation in the brain regions responsible for interoception and perception. Although OLP is primarily characterised by immune-mediated mucosal damage, it often has significant psychological comorbidity, particularly in the erosive form. Common features such as cortisol imbalance, disturbed cytokine patterns, and high levels of anxiety and depression suggest that these conditions may be due to overlapping systemic disorders. It is no longer sufficient to focus only on the visible lesions or symptom relief. Understanding these diseases/disorders through a more comprehensive psychoneuroendocrine immune system (PNEI) opens up new opportunities for early intervention, improved diagnostics, and more personalised therapeutic strategies that go beyond treating symptoms. Ultimately, these diseases/disorders require a more integrated and patient-centred approach, where understanding the whole system is as important as treating its individual parts.

## 1. Introduction

Oral mucosal diseases/disorders, such as burning mouth syndrome (BMS) and oral lichen planus (OLP), represent a complex interplay of biological, psychological, and social factors. Traditionally classified as localised or autoimmune pathologies, there is increasing evidence that these conditions may also reflect a broader dysregulation of the psychoneuroendocrine immune (PNEI) axis [1,2,3]. Although their clinical manifestations differ, both disorders are often associated with chronic psychological stress, altered hormonal status and immune dysfunction—highlighting the need for an integrative pathophysiological framework.

BMS is a chronic, idiopathic pain disorder that mainly affects perimenopausal and postmenopausal women. It is characterised by a persistent burning sensation of the oral mucosa without obvious clinical findings and is often accompanied by xerostomia, dysgeusia (metallic and bitter), and sleep disturbances [1,4,5]. The prevalence of BMS increases with age, and several studies have pointed to the role of concomitant psychological disorders such as anxiety, depression, and somatisation as exacerbating factors [2,6,7]. Neuroimaging and neurochemical studies have also implicated central sensitisation and the dysregulation of dopamine and serotonin signalling pathways in symptom persistence [1,3]. On the other hand, OLP is a chronic, T-cell-mediated inflammatory disease of the oral mucosa with an estimated prevalence of 1.0–2.0% in the general population. It manifests itself in various clinical forms, with the erosive form often causing considerable pain and discomfort [8,9]. In addition to its autoimmune aetiology, OLP is increasingly recognised as a psychosomatically influenced disease. An increased prevalence of anxiety, depression, and sleep disorders has been found in patients with OLP, particularly those with erosive lesions or chronic disease [3,9,10].

From a mechanistic perspective, chronic stress appears to play a central role in both BMS and OLP, as it activates the hypothalamic-pituitary-adrenal (HPA) axis, leading to increased cortisol levels and disruption of immune homeostasis [3,11,12]. Altered salivary cortisol concentrations, dehydroepiandrosterone (DHEA), and sex hormones such as oestrogen and testosterone have been observed in affected individuals, supporting the hypothesis that a neuroendocrine imbalance contributes to the pathophysiology of the disease [4,11,13]. In both diseases, inflammatory cascades—characterised by elevated levels of proinflammatory cytokines such as interleukin 6 (IL-6) and tumour necrosis factor alpha (TNF-α)—further exacerbate this vicious cycle of neuroimmune dysregulation [1,3,12].

Although the causal relationships are not yet fully understood, the convergence of neuronal, endocrine, and immunological disorders suggests that BMS and OLP may have overlapping biological substrates. From a clinical perspective, these diseases represent a significant burden on patients’ quality of life (QoL) and are difficult to treat with conventional local therapies alone. The discrepancy between somatic symptoms and objective findings often leads to diagnostic uncertainty and therapeutic frustration—both for patients and treating physicians. A deeper understanding of the neurobiological basis of BMS and OLP is essential for the development of multidisciplinary treatment approaches that go beyond symptom relief. However, there are still significant research gaps. It is still unclear to what extent psychological dysregulation is a cause or a consequence of these diseases. The temporal and mechanistic relationships between neuroendocrine biomarkers, cytokine profiles, and symptom severity have not yet been adequately characterised. Furthermore, there is limited evidence on the predictive value of salivary or hormonal biomarkers for disease progression or response to treatment.

In this narrative review, we aim to summarise the current state of knowledge on the psychoneurobiological mechanisms underlying BMS and OLP, focusing on the interactions between PNEI, and propose a multidimensional perspective that can guide future diagnostic and therapeutic strategies.

## 2. Materials and Methods

The aim of this study was to investigate the changes in PNEI factors in adults with BMS or OLP compared to healthy controls (Table 1).

Only systematic reviews (SRs) and meta-analyses (MAs) addressing the PNEI aspects of BMS and OLP were included in this narrative review. The studies had to fulfil the following criteria: the study addressed PNEI factors related to BMS or OLP; the studies used standardised criteria for the diagnosis of BMS or OLP, including clinical, histological, and/or laboratory diagnoses; it was an SR or MA; the publications were published in English before July 2025 (1983–2025) and were peer-reviewed. The literature was searched in the following electronic databases: PubMed/MEDLINE, Web of Science, Scopus, Cochrane Library, and Google Scholar. The search included the following keywords: “Burning Mouth Syndrome” AND “psychoneuroendocrinological aspects”, “Oral Lichen Planus” AND “psychoneuroendocrinological aspects”, “Meta-analysis” OR “Systematic Review”, “endocrine”, “immune system”, “psychological factors”, “stress”, “anxiety”, “depression”, “biomarkers”, “cortisol”, “saliva”.

We excluded studies that were not SR or MA; that did not include analyses of PNEI aspects; that focused only on clinical therapy without relevant biological or psychological parameters; that were not written in English; or that used inadequate diagnostic criteria for BMS or OLP. Two reviewers (D.M. and A.G.) independently screened the titles and abstracts of the studies to select those that met the inclusion criteria. In cases of discrepancies, a third reviewer (L.L.-M.) made the final decision. Data were then extracted according to a predefined protocol that included the following: type of study (SR, MA); biomarkers associated with BMS and OLP; psychological, neurological, endocrinological, and immunological factors; relationship between psychological and physiological parameters. The quality of the included SRs and MAs was assessed using the Cochrane Collaboration’s risk of bias tool for SRs and MAs, and each reviewer independently assessed the quality of the studies. Risk of bias included the following: study inclusion selectivity; reporting bias; methods of data analysis; outcome selection bias. The inclusion criterion for the review was moderate or low risk of bias.

Given the nature of the narrative review, an ethics committee was not required as no data with human participants were included. However, prior approval from the relevant ethics authorities was required for all included studies. As this is a narrative review, no formal meta-analysis was conducted. Instead, the focus is on a descriptive synthesis of the available evidence and the identification of gaps in the current literature that can form the basis for future studies.

The GenAI tool v4 [DALL•E (OpenAI)] was used to generate Figure 2 based on the textual description.

## 3. Results

**Figure 1:**
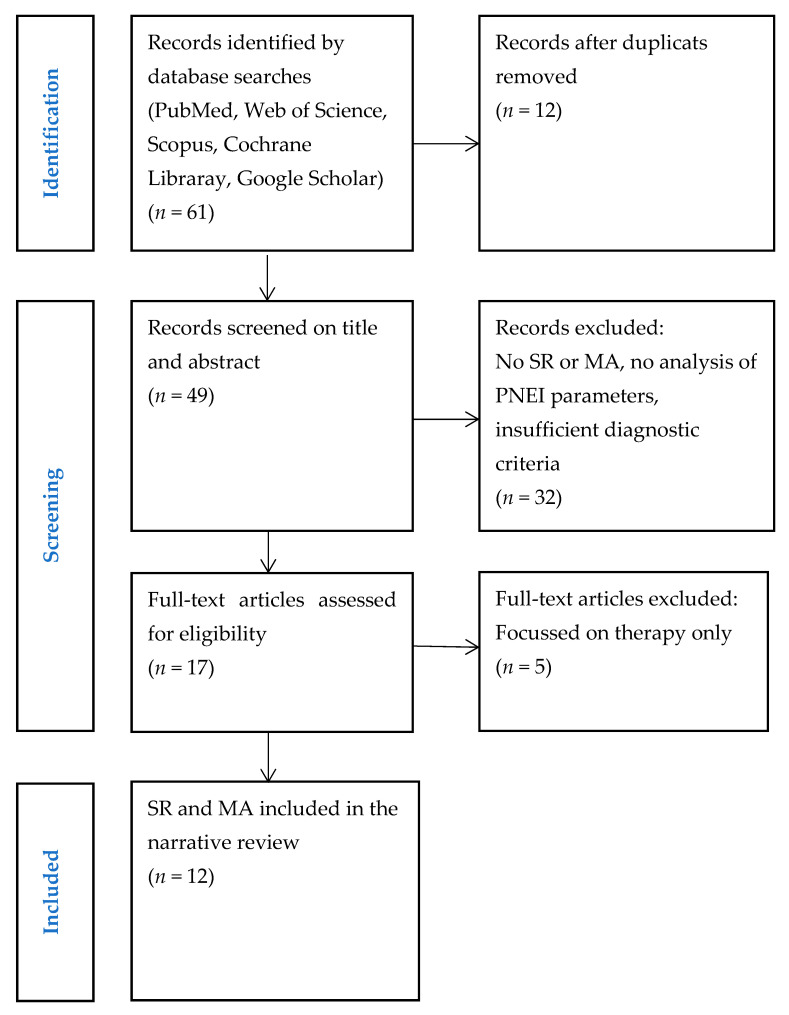
Study selection. Abbreviations: SR, systematic review; MA, meta-analysis; PNEI, psychoneuroendocrinoimmunology.

A search of electronic databases was conducted to find relevant SRs and MAs dealing with PNEI aspects of BMS and OLP. Additional records were identified by hand-searching the references of relevant articles. A total of 61 articles were found. After reviewing the titles and abstracts of 49 articles, 32 were excluded as they did not fulfil the inclusion criteria. Finally, 12 articles were included in this review. The selection process is illustrated in the PRISMA diagram (Figure 1).

The SR and MA by Galli F et al. showed that anxiety and depression are important psychological factors associated with BMS, and most of the included studies confirm the psychological component in the aetiopathogenesis of the disease [14]. The SR by Orliaguet M et al. concluded that the neuropathic and psychogenic components of BMS overlap and that the disease is best viewed through the concept of nociplastic pain [15]. In the SR and MA by Fernández-Agra M et al., 54 different salivary biomarkers were observed, of which only cortisol was included in the meta-analysis and showed a statistically significant higher concentration in BMS patients compared to control subjects (mean difference = 0.39; 95% CI [0.14–0.65]; *p* = 0.003) [12]. The SR and MA by He M et al. included 12 studies (43 salivary biomarkers and 35 serum biomarkers), of which only salivary cortisol concentration was statistically significantly higher in BMS patients and was associated with psychological symptoms [16]. The SR and MA by Kappes F et al. included 15 studies and showed a statistically significantly higher salivary cortisol concentration and immunoglobulin A (IgA) (mean difference = 0.53; 95% CI [0.33–0.74] and mean difference = 0.32; 95% CI [0.10–0.55], respectively) [17].

The SR by Cerqueira JDM et al. analysed 14 studies and showed that there is a significant correlation between mental disorders (stress, anxiety, depression) and the development of OLP [18]. The SR by Li K et al. also confirmed the association between OLP and mental health using a meta-analytical approach [19]. The SR and MA by De Porras-Carrique T et al. showed that OLP patients have a statistically significantly higher prevalence of depression, anxiety, and stress compared to the general population, which emphasises their importance in the clinical context [20]. The SR and MA by Mozaffari HR et al. found statistically significantly higher salivary and serum concentrations of interleukin 8 (IL-8) in OLP compared to healthy individuals (with significantly greater differences in saliva) [21]. The SR and MA by Koopaie M et al. identified specific salivary miRNAs (e.g., miR-27a, miR-137, miR-1290, miR-27b, miR-4484, miR-142, miR-1246) that accurately discriminate OLP patients from healthy individuals (sensitivity, ~0.80; specificity, ~0.89; AUC, 0.93) [22]. The SR and MA by Pires ALPV et al. found a higher salivary cortisol concentration (3.43 ng/mL) in OLP compared to control subjects (95% CI: 1.20–5.65), with high heterogeneity (I^2^ ≈ 98.9%) [23]. The SR and MA by De Porras-Carrique T et al. included 153 studies and showed a significant association between OLP and autoimmune diseases, particularly thyroid disease [prevalence ≈ 7.96%; odds ratio (OR) ≈ 1.99)] and diabetes mellitus (DM) (prevalence ≈ 9.41%; OR ≈ 1.64) [24].

The analysis suggests that further studies are needed that integrate psychological, neurological, endocrine, and immunological variables into a single protocol, i.e., develop and validate multifactorial models that include the above parameters. There is a need to standardise protocols for the collection and analysis of salivary biomarkers to allow replication and meta-analytical comparison of studies. Longitudinal studies are needed to observe the dynamics of biomarkers and psychological symptoms over time, especially before, during, and after treatment. Comparative studies analysing similarities and differences in the PNEI profiles of BMS and OLP are also needed to improve diagnostics and targeted therapy. Future studies also need to include genetic and epigenetic profiles that could explain the individual differences in stress sensitivity and immune dysregulation of BMS/OLP.

## 4. Discussion

### 4.1. Psychological Aspects of BMS and OLP

Psychological disorders are increasingly recognised as a central factor in both the clinical manifestation and underlying pathophysiology of BMS and OLP. Emotional dysregulation, chronic stress, anxiety, and depression not only exacerbate the perception of symptoms but may also play a role in the initiation, maintenance, and exacerbation of the disease. This complex interplay between psychological and somatic factors is underpinned by overlapping neurobiological mechanisms involving affective pain processing, cognitive appraisal, and neuroimmune signalling. Epidemiological data clearly demonstrate the high prevalence of psychological comorbidities in both diseases. A comprehensive SR and MA of over 6800 OLP patients revealed that anxiety occurs in over 54.0% of patients, while depression and stress occur in approximately 31.0% and 41.0% of patients, respectively. Compared to healthy controls, OLP patients were significantly more likely to suffer from depression (OR = 6.15), anxiety (OR = 3.51), and stress (OR = 3.64). Emotional stress was particularly pronounced in patients with the erosive subtype of OLP, where higher clinical severity correlated with a poorer psychological profile [20].

**Figure 2:**
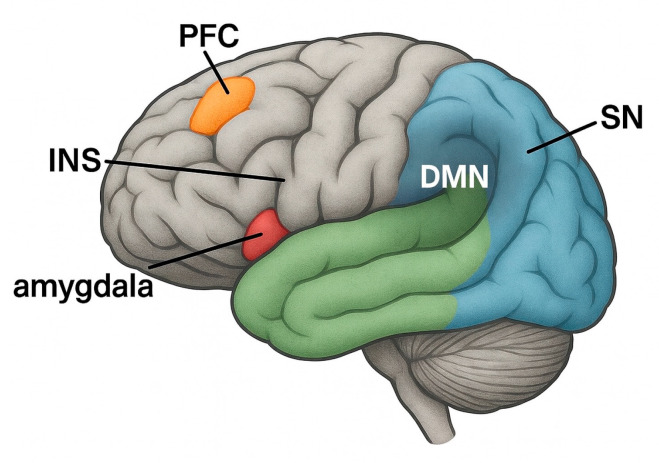
Brain regions involved in the perception and regulation of pain in chronic oral diseases/disorders such as burning mouth syndrome (BMS) and oral lichen planus (OLP). Highlighted areas include the prefrontal cortex (PFC), anterior cingulate cortex (ACC), insula (INS), amygdala, and two functionally relevant brain networks: the default mode network (DMN) and the salience network (SN). Figure 2 shows a lateral view in which representative regions of the DMN and SN are schematically labelled. These structures are involved in cognitive–affective processing, attention to pain stimuli, interoception, and the modulation of nociceptive input—mechanisms that underlie chronic orofacial pain syndromes. Abbreviations: BMS, burning mouth syndrome; OLP, oral lichen planus; PFC, prefrontal cortex; ACC, anterior cingulate cortex; INS, insula; DMN, default mode network; SN, salience network.

Similar tendencies can also be observed with BMS. Numerous studies have reported that BMS patients often have increased levels of psychological distress, including anxiety, depressive symptoms, sleep disturbances, and cognitive impairment. The SR by Dibello V et al. confirmed that BMS patients, especially middle-aged and older adults, are significantly more likely to suffer from depressive and anxiety disorders than the general population [2]. This psychological distress may not only be a response to chronic pain but rather a bidirectional relationship in which affective disorders exacerbate nociceptive processing, leading to the persistence of symptoms and resistance to conventional treatment [5]. Neuroimaging studies provide converging evidence of altered brain activity in regions that play a role in both pain and emotion. BMS patients show changes in the anterior cingulate cortex (ACC), insular cortex, prefrontal cortex (PFC), and amygdala—areas that link nociceptive input to emotional and cognitive appraisals [15]. The insular cortex in particular plays a crucial role in interoception—the brain’s ability to monitor internal body states. Altered interoceptive processing in BMS can lead to over-perceiving oral sensations and misinterpreting them as painful, contributing to a state of increased symptom severity [25]. Furthermore, dysfunction in intrinsic brain networks such as the default mode network (DMN) and salience network (SN) has been proposed as the neurobiological basis for the affective amplification of pain in chronic orofacial disease (Figure 2). The DMN, which is involved in self-focused thinking and basic mental activities, may contribute to excessive rumination and catastrophising, while the SN, particularly the anterior insula and ACC, controls the shift in attention to emotionally meaningful stimuli—including pain. Aberrant activity in these networks has been found in both chronic pain and mood disorders and may serve as a neurocognitive bridge between BMS and comorbid psychological disorders [26].

It is important to point out that there are qualitative differences in the emotional profiles of BMS and OLP patients. In OLP, the emotional disturbance is often reactive—a consequence of visible lesions, chronic immune-mediated inflammation, or fear of malignant transformation. In contrast, the psychological disturbance in BMS is more functional and intrinsic, with symptoms such as burning in the mouth occurring without a clear tissue pathology and often accompanied by “emotional amplification” and maladaptive interoceptive perception [27]. From a clinical perspective, these psychological dimensions justify active screening and targeted interventions. Validated instruments such as the Hospital Anxiety and Depression Scale (HADS), the Beck Depression Inventory (BDI), and the Perceived Stress Scale (PSS) can help with early detection. In addition, cognitive behavioural therapy (CBT), mindfulness-based stress reduction, and pharmacotherapy with selective serotonin reuptake inhibitors (SSRIs) have been shown to be effective in improving both psychological well-being and oral symptom burden in BMS and OLP patients [28]. Integrating psychological interventions into multidisciplinary treatment models may therefore lead to better outcomes than symptom-focused dental or pharmacological treatment alone. Recent clinical findings support the efficacy of non-pharmacological interventions in BMS. The SR by Tan X et al. showed that psychological treatments, particularly CBT and mindfulness-based approaches, significantly reduced pain intensity and psychological distress in BMS patients. These interventions not only improved short-term symptoms but also contributed to sustained functional and emotional recovery, emphasising the role of psychological support in integrated care models [29].

The results indicate that psychological factors are not an epiphenomenon but an essential component of the pathogenesis and clinical course of BMS and OLP. This underlines the need for integrated diagnostic and therapeutic strategies that include psychological screening, stress management, and behavioural interventions in addition to local and systemic therapies. Understanding the affective dimensions of these diseases can improve patient outcomes and reduce the chronicity of the disease.

### 4.2. Neurological Aspects of BMS and OLP

The neurological basis of BMS and OLP reflects complex interactions between peripheral sensory input and central nociceptive processing. Although BMS is classically considered a neuropathic pain disorder and OLP is considered an immune-mediated mucosal disease, both diseases share common features, such as altered sensory transmission, central sensitisation, and neuroplastic mismatch.

#### 4.2.1. Peripheral Sensory Dysfunction

The trigeminal nerve, particularly its maxillary and mandibular branches, is responsible for the sensory innervation of the oral mucosa. In BMS, studies have demonstrated subtle damage or dysfunction in small, unmyelinated C-fibres and thinly myelinated A-delta fibres, resulting in altered pain transmission without an obvious nerve lesion visible on imaging or clinical examination [30]. Quantitative sensory testing (QST) has shown that thermal and mechanical sensation thresholds are impaired in the affected mucosa of BMS patients, particularly at the tip of the tongue and anterior palate [31]. These findings indicate the presence of a peripheral neuropathic component, even if it is often subclinical.

Although OLP is not primarily neurological in nature, it can also cause secondary sensory nerve fibre dysfunction due to chronic inflammation, particularly in erosive or ulcerative forms. A persistent inflammatory insult mediated by T-cell infiltrates and cytokine release can sensitise neighbouring nociceptive afferents and contribute to hyperalgesia or allodynia [32].

#### 4.2.2. Central Sensitisation and Nociplastic Pain

Chronic pain in BMS is increasingly recognised as a form of nociplastic pain—pain that is due to altered central nociceptive processing without clear peripheral input or nerve damage [33]. Central sensitisation mechanisms include increased excitability of second-order neurons in the spinal trigeminal nucleus (STN) and impaired descending inhibitory pathways from the periaqueductal grey (PAG) and rostral ventromedial medulla (RVM). Functional neuroimaging studies show increased activation in the ACC, insular cortex, and somatosensory areas, indicating abnormal central pain integration and top-down modulation [34]. Importantly, BMS patients often show neuroplastic changes, including a reduction in grey matter volume in pain-related cortical areas and altered functional connectivity within pain networks [26]. These changes indicate a long-standing maladaptive reorganisation of sensory circuitry that is likely maintained by continuous pain perception in the absence of somatic pathology.

OLP, on the other hand, shows no primary features of central sensitisation in most patients. However, in severe or chronic erosive/ulcerative cases, the persistent nociceptive input may lead to secondary central hyperexcitability, especially if the pain persists beyond the typical inflammatory episodes. Indeed, OLP patients who develop chronic orofacial pain without visible lesions may develop a similar phenotype to BMS patients, with overlapping central mechanisms. It is important to emphasise that these overlaps are limited to specific populations, i.e., they are not universal.

#### 4.2.3. Trigeminal Pathway and Brain Regions Involved

The central processing of orofacial pain involves multiple relays along the trigeminal pathway, beginning with peripheral afferents that project into the STN. From there, second-order neurons ascend via the trigeminothalamic tract to the ventral posteromedial nucleus (VPM) of the thalamus and finally project to cortical areas such as the primary and secondary somatosensory cortex (S1, S2), the insula (INS), and the ACC [35]. In BMS, abnormal activity in these regions was confirmed by functional Magnetic Resonance Imaging (fMRI) and Positron Emission Tomography (PET) studies, which showed increased spontaneous activity and impaired connectivity within the pain matrix. INS and ACC are particularly involved in the affective–motivational aspects of pain, while the somatosensory cortexes mediate the sensory-discriminative components. A dysregulation in this system could explain why BMS patients perceive pain although there is no clear peripheral nociceptive drive [34].

#### 4.2.4. Descending Modulatory Systems

Descending pain modulatory pathways play a crucial role in controlling nociceptive input at the spinal and brainstem levels. These circuits involve the PFC, amygdala, hypothalamus, and brainstem centres such as the PAG and RVM. In healthy people, these systems maintain a balance between pain relief and pain inhibition. In BMS, this balance appears to be disturbed, as there is evidence of impaired endogenous inhibition and increased descending facilitation [36]. It is hypothesised that such disruption contributes to persistent pain despite the absence of persistent tissue damage. Furthermore, the interaction between emotional states and these modulating systems is crucial. Negative affective states such as anxiety and depression can reduce descending inhibition and promote central sensitisation. This emphasises the close relationship between the psychological and neurological dimensions of BMS.

### 4.3. Endocrine Aspects of BMS and OLP

The endocrine system—in particular, the sex hormones and the HPA axis—plays an important role in the pathogenesis and symptoms of BMS and OLP. Endocrine dysregulation, which is often underestimated, contributes to nociceptive sensitivity, mucosal homeostasis, and immune–neuroendocrine crosstalk, especially in female-dominated disorders such as BMS.

#### 4.3.1. Role of Sex Hormones and Oestrogen Deficiency

BMS has a marked gender-specific predilection, with the incidence being highest in perimenopausal and postmenopausal women. This epidemiological pattern has led to hypotheses about the role of oestrogen deficiency in modulating the sensation of the oral mucosa. Oestrogens affect peripheral nerve function, neuroinflammation, and expression of receptors, including transient receptor potential vanilloid 1 (TRPV1), which is involved in heat and pain signalling in the oral mucosa [37]. A reduction in circulating oestradiol levels may contribute to decreased salivary secretion, altered oral epithelial trophism, and increased pain sensitivity. Some studies have shown altered oestrogen receptor expression in the oral mucosa of BMS patients, although the results are contradictory. Experimental models support the hypothesis that oestrogen deprivation enhances thermal hyperalgesia via peripheral sensitisation mechanisms [30].

In OLP, the difference between the sexes is not as great as in BMS, but there is evidence that sex hormones may influence immunomodulation and epithelial integrity. Oestrogens can modulate T cell activity, cytokine profiles (e.g., IL-6 and TNF-α), and keratinocyte behaviour, which may influence disease onset and the severity of disease flares in hormonally dynamic states [38].

#### 4.3.2. HPA Axis Dysregulation and Cortisol Imbalance

It is known that chronic pain and psychological stress dysregulate the HPA axis and lead to altered cortisol dynamics. In BMS patients, studies have shown inconsistent but notable changes in salivary and plasma cortisol concentrations, suggesting a blunted or hyperactive stress response [39]. Some patients show increased cortisol concentrations in the evening or a flattened diurnal fluctuation—both signs of impaired neuroendocrine feedback. This imbalance can impair central pain modulation, weaken mucosal immunity, and promote systemic inflammation. In addition, cortisol interacts with neurotransmitter systems (e.g., serotonin and noradrenaline) that influence mood and pain perception. This may partly explain the overlap between endocrine, psychological, and neurological dysfunction in BMS.

In OLP, chronic inflammation may act as a long-term stressor leading to persistent activation of the HPA axis. Elevated serum cortisol concentrations have been observed in patients with erosive OLP, particularly those with comorbid anxiety or depressive symptoms. This bidirectional relationship suggests that neuroendocrine abnormalities are not only a consequence of chronic inflammation but also a driver of immune system dysregulation. The case–control study by Glavina A et al. was the first to compare the psychoendocrinological profile of BMS or OLP patients. The authors showed that BMS patients had higher salivary cortisol concentrations compared to OLP patients and control subjects, but without statistical significance (0.52 vs. 0.44 vs. 0.44 µg/dL; *p* = 0.31) [40].

#### 4.3.3. Endocrine–Immune–Neural Interactions

The endocrine system exerts regulatory control over the functions of the immune and nervous systems via hormone receptors and indirect feedback mechanisms. For example, glucocorticoids modulate the response of T helper cells and cytokine production, while oestrogens can suppress Th1-type inflammation and improve the function of regulatory T cells (Treg) [38]. In BMS and OLP, disruption of this balance can lead to a state of chronic low-grade inflammation and sensory hypersensitivity, particularly in individuals with comorbid emotional stress. In addition, hormonal fluctuations can exacerbate mucosal barrier dysfunction and neuroinflammatory signalling pathways, increasing the chronicity of symptoms. These interactions highlight the importance of considering endocrine status in both diagnostic and therapeutic strategies. Although hormone supplementation remains controversial, selected BMS patients may benefit from systemic hormone therapy or centrally acting modulators that indirectly restore neuroendocrine balance.

### 4.4. Immunological Aspects of BMS and OLP

OLP and BMS are two different orofacial diseases with overlapping symptoms but distinctly different immunopathogenic mechanisms. OLP is primarily an autoimmune, T-cell-mediated inflammatory disease, whereas BMS was thought to be an idiopathic or neuropathic disorder. However, recent findings suggest that low-grade immune activation and neuroimmune interactions may also play a role in the pathogenesis of BMS. In both diseases, the dysregulation of the immune system not only leads to the development of symptoms but also influences tissue homeostasis and pain perception.

#### 4.4.1. OLP as a T-Cell-Mediated Autoimmune Disease

The immunopathology of OLP is characterised by a chronic, cytotoxic T-cell reaction directed against basal keratinocytes of the oral mucosa. CD8+ T lymphocytes infiltrate the epithelial–connective tissue interface and induce keratinocyte apoptosis via perforin, granzyme B, Fas/FasL, and TNF-α signalling pathways [41]. This immune-mediated damage leads to subepithelial separation, the formation of Civatte bodies, and epithelial degeneration—the typical features of OLP histopathology. In addition, CD4+ T cells and Th1-related cytokines [e.g., interferon gamma (IFN-γ), interleukin 2 (IL-2), TNF-α] are increased in the lesional tissue and saliva of OLP patients. More recently, Th17 cells and interleukin 17 (IL-17) have also been detected, particularly in erosive subtypes [42]. The presence of Treg cells also contributes to uncontrolled inflammation, a loss of immune tolerance, and chronicity [43]. OLP also involves the activation of antigen-presenting cells (APCs), with an upregulation of Major Histocompatibility Complex (MHC) class II and costimulatory molecules on dendritic cells (DCs) and Langerhans cells. The source of the triggering antigen is still unclear—viral [e.g., Hepatitis C Virus (HCV)], bacterial, dental materials, or self-antigens of altered keratinocytes are possible [44].

#### 4.4.2. Neuroimmunological Interactions in BMS

Although BMS is not a classic inflammatory disease, recent evidence suggests that neuroimmune interactions and low-grade inflammation play a role in a subset of patients. Elevated salivary concentrations of proinflammatory cytokines such as IL-6, IL-8, and TNF-α have been found in BMS patients compared to healthy controls, especially in patients with high psychological stress or comorbid psychiatric symptoms [45]. These cytokines are able to sensitise peripheral nociceptors and disrupt local epithelial integrity. Furthermore, glial activation in the trigeminal ganglion and brainstem, as demonstrated in experimental models of neuropathic oral pain, suggests that immune-like glial responses may enhance nociceptive signalling at the central level. Microglia and astrocytes, when activated by cytokines or neuropeptides, release additional proinflammatory mediators [e.g., interleukin 1 beta (IL-1β) and nitric oxide (NO)] that further enhance pain transmission [46]. These neuroimmune mechanisms are bidirectional: stress and depression can enhance immune activation, while cytokines can modulate mood and cognition by influencing serotonin and glutamate pathways. This feedback loop may be the cause of the co-occurrence of pain, emotional dysregulation, and oral mucosal symptoms in BMS.

Recent findings on neuroimmune interactions also emphasise the activation of microglia and astrocytes in the trigeminal system as important amplifiers of orofacial pain. Proinflammatory cytokines released by these glial cells—such as IL-1β and TNF-α—can sensitise central nociceptive neurons and impair descending inhibitory circuits. This feedback loop between immunological and neuronal elements may be a common mechanism underlying chronic pain in both BMS and severe forms of OLP [47].

#### 4.4.3. Salivary Biomarkers and Inflammatory Mediators

Several studies have investigated salivary biomarkers as potential non-invasive tools to assess immune status in both BMS and OLP. In OLP, elevated levels of IL-6, IL-17, interleukin 23 (IL-23), TNF-α, and matrix metalloproteinase-9 (MMP-9) have been associated with active disease, particularly in erosive forms [48]. In BMS, the presence of IL-8 and substance P in saliva correlates with pain intensity and burning sensation, suggesting local immune activation and the involvement of neuropeptides [5]. The study by Glavina A et al. showed that there was no statistically significant difference in salivary IL-6 concentration between OLP patients, BMS patients, and control subjects (*p* = 0.244). Taking into account all the shortcomings of the study, OLP patients had a slightly higher salivary IL-6 concentration compared to BMS patients (4.35 vs. 3.42), indicating a dominant inflammatory pathogenesis of OLP and a loss of neuroprotection in BMS [49].

### 4.5. Integrated PNEI Perspective on BMS and OLP

As already mentioned, BMS and OLP are two diseases with different aetiopathogenetic mechanisms (neuropathic vs. autoimmune). However, their pathophysiological mechanism is much more complex and includes psychological, neurological, endocrine, and immunological aspects (Table 2). An integrated PNEI approach (multidisciplinary) is required for both diseases.

## 5. Conclusions

The psychological, neurological, endocrine, and immunological mechanisms underlying BMS and OLP together form a closely linked biopsychosocial model. Both diseases are not limited to isolated areas—such as mucosal inflammation or peripheral neuropathy—but involve systemic dysregulation on multiple physiological axes. BMS is increasingly recognised as a disease of central pain amplification and affective modulation in which psychological stress, neuroplastic changes, hormonal imbalances, and subtle immune activation interact to cause chronic oral discomfort without visible lesions. In contrast, OLP is a prototypical autoimmune mucosal disease, but its course is strongly influenced by psychological stress, neuroimmune interactions, and endocrine changes, especially in hormonally sensitive populations.

The current diagnosis of BMS and OLP is difficult, as there are no standardised international diagnostic criteria for either clinical entity. The diagnosis of BMS is further complicated by the lack of objective diagnostic tools, i.e., diagnosis is by exclusion. This contributes to a bias in the study results and makes it impossible to draw relevant/objective clinical conclusions. As mentioned above, the association of BMS (stronger) and OLP with mental disorders is well known, i.e., they are part of the multifactorial aetiology of these diseases/disorders. Standardised diagnostic instruments must be used to determine the relationship between BMS and OLP with mental disorders. OLP also lacks a comprehensive instrument to distinguish between different clinical forms of the disease, which contributes to heterogeneity. Based on the PNEI approach, the diagnostic limitations of BMS are the lack of clear diagnostic criteria, the difficulties in quantifying psychological and emotional factors, the overlap of BMS symptoms with other clinical entities, and the multidimensionality of the disorder (no comprehensive diagnostic framework for each component). The therapeutic limitations of BMS are a comprehensive and individualised therapeutic approach (ineffectiveness of standard therapies), the association with chronic stress, hormonal and immunological imbalance, the complexity of causes, i.e., their overlap, and the lack of specialised PNEI therapists. The diagnostic limitations of OLP are the lack of specific biomarkers for diagnosis, psychosocial factors (cause or secondary effect), multifactorial aetiology (synergistic effect of genetic, immunological, and environmental factors), and the limited use of specific tests for its detection. The therapeutic limitations of OLP are the inconsistent response to therapy, the significant side effects of long-term treatment with corticosteroids and immunosuppressants, the unavailability or inadequate use of stress therapies (CBT), hormonal imbalance (risks of long-term treatment with hormone replacement therapies), and the lack of specialised PNEI therapists (the therapeutic approach is often difficult due to the presence of other chronic diseases). BMS and OLP require a careful diagnosis, an individualised approach, and a multidisciplinary team (dentists, dermatologists, immunologists, psychologists) to achieve a favourable therapeutic effect.

The overlapping symptoms and common risk factors between these diseases argue in favour of a unified conceptual framework. Disorders of interoceptive perception, central sensitisation, HPA axis regulation, and T-cell-mediated inflammation form a pathophysiological continuum rather than separate silos. From a clinical perspective, this integrated view emphasises the importance of multidisciplinary treatment strategies. Optimal treatment should include not only local and systemic medical interventions but also psychotherapeutic support, stress regulation, neuroendocrine assessment, and immune monitoring. Recognising the interdependence of these systems may ultimately improve diagnostic accuracy, enhance treatment outcomes, and reduce the risk of chronicity in both BMS and OLP.

## Figures and Tables

**Table 1:** The PICO of this study.

P (Population)	Adults with BMS or OLP
I (Intervention)	PNEI factors (e.g., psychological stress, neuropeptides, hormonal status, cytokines)
C (Comparison)	Healthy controls
O (Outcome)	Correlations or differences between stress levels, immunological parameters, and biomarkers that influence the pathogenesis and clinical manifestations of BMS or OLP

Abbreviations: PICO, population, intervention, comparison, outcome; BMS, burning mouth syndrome; OLP, oral lichen planus; PNEI, psychoneuroendocrinoimmunology.

**Table 2:** Comparative PNEI aspects of BMS and OLP.

Domain	BMS	OLP
Psychological	High anxiety, depression, emotional amplification; altered activity of DMN and SN	Reactive stress due to visible lesions and chronicity; fear of malignant transformation
Neurological	Peripheral C- and Aδ-fibre dysfunction; central sensitisation; neuroplasticity involving ACC and INS	Secondary sensitisation in erosive forms; no primary neuropathic mechanism
Endocrine	Oestrogen deficiency (peri/postmenopause); HPA axis dysregulation; abnormal cortisol rhythm	Oestrogen influences T-cell activity; chronic inflammation influences cortisol dynamics
Immunological	Low-grade inflammation (↑ IL-6, IL-8, TNF-α); glial activation and neuroimmune crosstalk	T-cell-mediated autoimmunity (↑ IFN-γ, IL-6, IL-17; ↓ Treg cells)
Clinical features	Burning sensation without visible lesions; typically affects the tongue, lips, and hard palate; often bilateral	White reticular or erosive lesions; may be painful or cause burning; usually symmetrically distributed
Therapeutic approach	CBT, mindfulness, SSRIs, neuromodulators (e.g., clonazepam); multidisciplinary care model	Topical/systemic corticosteroids and immunomodulators; optional psychological support

Abbreviations: PNEI, psychoneuroendocrine immunity; BMS, burning mouth syndrome; OLP, oral lichen planus; DMN, default mode network; SN, salience network; ACC, anterior cingulate cortex; INS, insula; IL-6, interleukin 6; IL-8, interleukin 8; TNF-α, tumour necrosis factor alpha; IFN-γ, interferon gamma; IL-17, interleukin 17; Treg, regulatory T cells; CBT, cognitive behavioural therapy; SSRIs, selective serotonin reuptake inhibitors.

## Data Availability

Not applicable.

## Abbreviations

The following abbreviations are used in this manuscript:

BMS	burning mouth syndrome
OLP	oral lichen planus
PNEI	psychoneuroendocrine immune system
HPA	hypothalamic–pituitary–adrenal axis
DHEA	dehydroepiandrosterone
IL-6	interleukin 6
TNF-α	tumour necrosis factor alpha
QoL	quality of life
SR	systematic review
MA	meta-analysis
PRISMA	preferred reporting items for systematic reviews and meta-analyses
CI	confidence interval
IgA	immunoglobulin A
IL-8	interleukin 8
miRNA	microRNA
AUC	area under the curve
OR	odds ratio
DM	diabetes mellitus
ACC	anterior cingulate cortex
PFC	prefrontal cortex
DMN	default mode network
SN	salience network
INS	insula
HADS	hospital anxiety and depression scale
BDI	beck depression inventory
PSS	perceived stress scale
CBT	cognitive behavioural therapy
SSRIs	selective serotonin reuptake inhibitors
QST	quantitative sensory testing
STN	spinal trigeminal nucleus
PAG	periaqueductal grey
RVM	rostral ventromedial medulla
VPM	ventral posteromedial nucleus
fMRI	functional magnetic resonance imaging
PET	positron emission tomography
TRPV1	transient receptor potential vanilloid 1
Treg	regulatory T cells
IFN-γ	interferon gamma
IL-2	interleukin 2
IL-17	interleukin 17
APCs	antigen-presenting cells
MHC	major histocompatibility complex
DCs	dendritic cells
HCV	hepatitis C virus
IL-1β	interleukin-1 beta
NO	nitric oxide
IL-23	interleukin 23
MMP-9	matrix metalloproteinase-9
